# Impact of cold exposure on shift working seafood handlers in Northern Norway: a comparative analysis across work shifts

**DOI:** 10.1186/s12995-025-00469-2

**Published:** 2025-06-23

**Authors:** Phong K. T. Chau, Tiril Schjølberg, Mina Baarnes Eriksen, Anne-Mari Gjestvang Moe, Pål Graff, Fred Haugen

**Affiliations:** 1 Division of Work Psychology and Physiology, National Institute of Occupational Health (STAMI), Pb 5330 Majorstuen 0304, Oslo, Norway; 2 Division of Occupational Chemistry, National Institute of Occupational Health (STAMI), Oslo, Norway; 3 Faculty of Medicine, University of Oslo, Oslo, Norway

**Keywords:** Shift work, Cold exposure, Breaks, Occupational health, Thermoregulation

## Abstract

**Objective:**

This study aimed to investigate the impact of occupational thermal exposure on shift workers, specifically whether cold exposure elicits distinct physiological responses and thermoregulatory recovery across different tasks and shift types.

**Methods:**

Observational study at two factories processing prawns in Northern Norway in which 32 shift-working seafood handlers with different task responsibilities were followed for a single shift (morning, evening, night). The participants answered questionnaires regarding thermal exposures at work and related symptoms; these were compared to answers from 12 administration workers. Personal thermal loggers measured the range of temperature exposures associated with four different seafood handler work tasks. Pre- and post-shift plasma levels of FGF21, GDF15 and cytokines were analysed using immunoassays. As a proxy for thermoregulatory response across different shift types, hand temperature was measured repeatedly before and after breaks using a thermal imaging camera.

**Results:**

Most seafood handlers reported subjective impact from cold exposure. Cold working conditions of ≤ 10 ℃ were measured across all shifts and three different seafood handling tasks. The morning shift—seafood handlers displayed lower plasma FGF21 post-shift vs. pre-shift; the evening and night shifts showed no difference. GDF15 levels remained unchanged regardless of shift types but were positively correlated with age. Night shift was associated with increased plasma IL6 post-shift vs. pre-shift. Thermoregulatory responses showed a positive linear relationship with break duration but did not differ between shifts.

**Conclusions:**

The findings suggest that exposure levels are closely linked to specific tasks and shifts, with thermoregulatory responses varying by task type and time of day.

**Supplementary Information:**

The online version contains supplementary material available at 10.1186/s12995-025-00469-2.

## Introduction

The seafood industry employs over 56 million people worldwide [1] and 86,000 individuals in Norway [2]. Cold exposure [3–5] and shift work [6–8] emerge as prominent factors that may significantly impact the health and well-being of seafood handlers.

Shift work is common in the industry and generally refers to any work schedule that falls outside the standard 09:00 to 17:00 daytime hours [9, 10]. More specifically, it includes any work occurring outside the 07:00 to 18:00 window for durations exceeding six hours, and encompasses night shifts, rotating shifts, and split shifts [11]. Shift work may disrupt the body's natural circadian rhythm [12], and it can lead to various adverse health outcomes, including sleep disturbances [7, 13]. Night shift work has also been associated with cardiovascular diseases, obesity, digestive problems, and diabetes [8].

Seafood processing environments are characterized by cold temperatures as products must be stored, processed, and packaged in chilled or freezing conditions to maintain freshness and safety [14]. A cold working environment is defined as an environment with an ambient temperature at or below 10 ℃ [15]. Extended exposure to a low temperature working environment might have adverse implications if no sufficient measures are in place [16–18]. When the skin is exposed to a low temperature, superficial capillaries will constrict to reduce blood flow and maintain the body temperature [19]. Continuous cold exposure to the skin causes localized hypothermia, where excessive vasoconstriction reduces warm blood flow to the exposed body part and thus decreases the tissue temperature [19]. Moreover, exposure to cold reduces skin temperature, negatively affecting overall dexterity and tactile sensitivity [20]. This impairment significantly heightens the risk of operational failures and subsequent cold-related injuries [21, 22]. In addition, the hands and fingers are particularly susceptible to cold injuries [23–26] and to a loss of manual dexterity due to cold-induced vasoconstriction [27].

While cold exposure and shift work are each recognised as significant occupational stressors [3–8], their combined effects on worker physiology remain insufficiently explored, particularly in industries like seafood processing where both are integral to the job. Importantly, shift work disrupts the body’s internal circadian clock, which regulates immune function [28], hormone secretion [29], and thermoregulation [30]. This disruption may not only impair recovery from cold stress but also alter physiological responses such as cytokine production [31], skin temperature regulation [32], and thermogenic hormone release [33]. Because many of these responses may follow circadian rhythms, cold exposure during night shifts or at biologically misaligned times may result in exaggerated or blunted physiological effects compared to daytime exposure [34]. Thus, understanding how cold impacts shift workers across times of day is important for developing targeted workplace health strategies.

Interleukin (IL)6, IL1β, and tumour necrosis factor (TNF) are key pro-inflammatory cytokines that play essential roles in the immune response. These factors display a diurnal pattern, with levels fluctuating throughout the day, and disruptions to circadian rhythms, such as those caused by shift work, can alter secretion, potentially amplifying inflammatory responses [35]. Prior studies examining the impact of cold exposure on inflammatory markers have demonstrated varying results: Brenner et al. found limited or no immunological changes [36]; other studies have found reduced cytokine production in response to cold air [37], whereas Rhind et al. reported that cold exposure increased the expression of IL-6 while decreasing the expression of IL1β and TNF [38]. Fibroblast growth factor 21 (FGF21) and growth differentiation factor 15 (GDF15) are hormones activated in response to cold stress [34, 39, 40]. They have a crucial function in maintaining energy balance and regulating the metabolism of substances in rodents [39] and humans [41, 42]. FGF21 and GDF15 stimulate a thermogenic mechanism to regulate thermal equilibrium in rodents. Yet, the impact of cold exposure on plasma levels of FGF21 and GDF15 in cold-exposed workers has not been well investigated.

This study aimed to assess the effects of occupational thermal exposure in shift work, with a particular focus on whether cold exposure elicits distinct physiological responses at different times of the day. To achieve this, we investigated the impact of cold exposure and subsequent recovery during breaks among shift-working seafood handlers in Northern Norway.

## Methods

### Study participants and setting

**Fig. 1:**
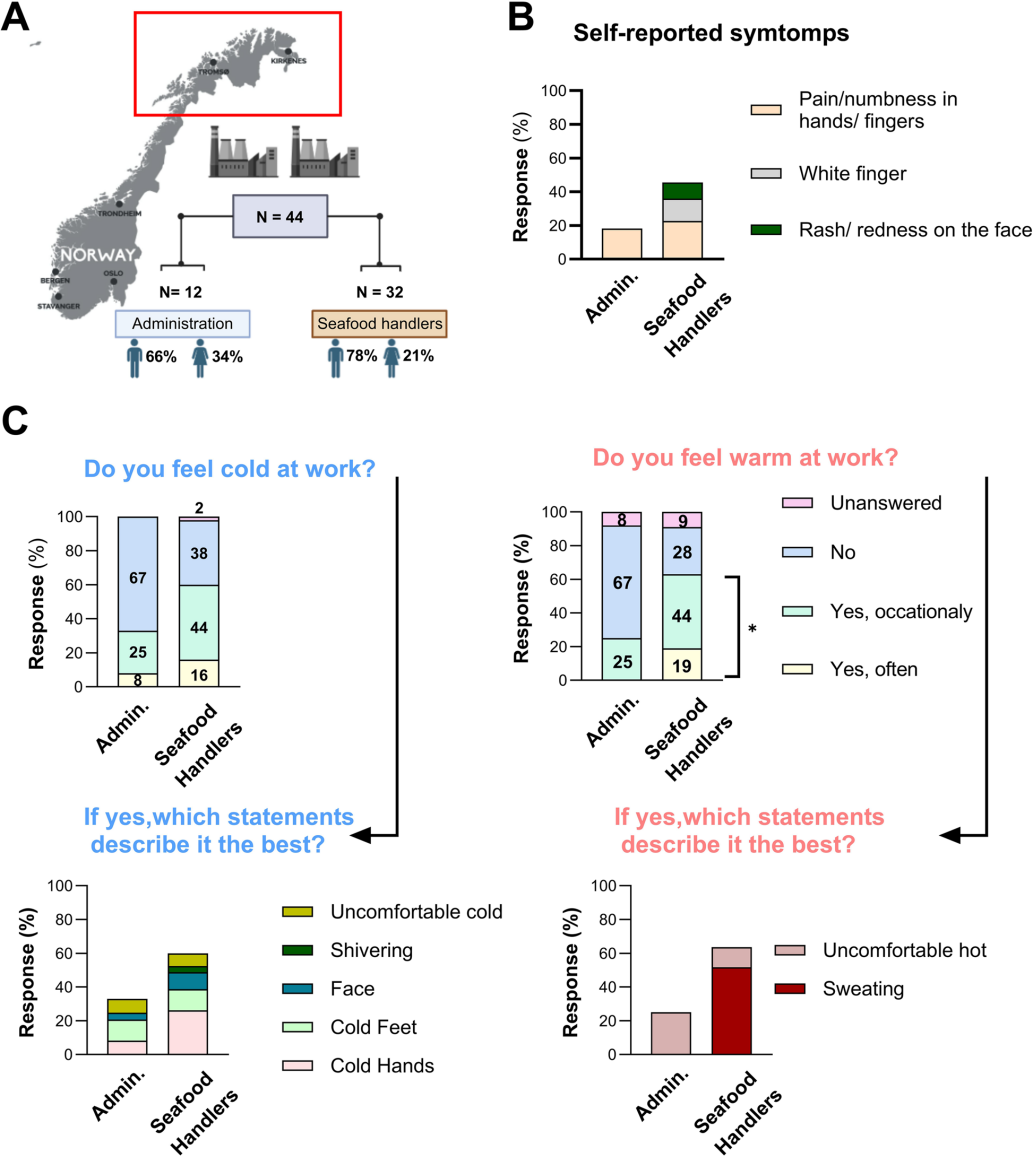
The participants'characteristics and their questionnaire responses. **A** The study was conducted in two seafood factories in Northern Norway with a total of 32 shift-working seafood handlers participating. For the questionnaire, an additional 12 non-shift day-working personnel participated from the same factories. **B** Cold exposure-related symptoms were assessed using a questionnaire. **C** Each participant was asked to respond to questions about their perception of the ambient working environment, specifically whether they perceive it as cold or warm. If the participants responded affirmatively to these questions, they were presented with the follow-up questions. Statistical significance was tested by Chi-square test; *P < 0.05. Data is shown as the percentage

A cross-sectional observational field study was conducted at two seafood factories processing prawns in Northern Norway (Fig. 1A). At these sites, shift-working seafood handlers were recruited for participation in the study (*N* = 32). Self-reported characteristics of the seafood handlers study sample are presented (Table 1). The data collection was performed on-site on May 10 to 11–2022 (plant 1) and June 14 to 15–2022 (plant 2), for approximately 24 h, covering the morning shift, evening shift and the night shift. Thus, the data for each individual represent a snapshot of the specific shift they worked on the day of measurement, enabling comparisons across independent samples of shift workers on morning, evening, and night shifts. In order to capture task-specific differences, seafood handlers were classified into four groups according to their primary job functions: 1) Thawers: perform work related to the process of defrosting frozen prawns to prepare them for further handling; 2) Operators: operate the actual processing machinery which peels, cooks, sorts, and packs prawns; 3) Controllers: work close to the production to monitor and manage the entire process to ensure quality, safety, and efficiency; and 4) Packers: sort the seafood product into suitable packaging, label and securely seal the packages before placing them in cold storages to await further transport to retailers. For selected measurements, the blue-collar sample was also compared with a white-collar reference group consisting of administration workers at the same two factories (*N* = 12). The administration workers performed their tasks in an office environment and did not work shifts. Shifts were partially overlapping and of different durations; there were variations in the start and end times of morning, evening, and night shifts. In this study, startups from 06:00–10:00 were treated as morning shift; startups from 14:00–16:00 were treated as evening shift; startups from 19:00–23:00 were treated as night shift. The seafood handlers wore insulative protective clothing including gloves.

**Table 1:** The characteristics of seafood handlers included in the study

Shift studied	Shift working seafood handlers (*N* = 32)		
	Morning	Evening	Night
Age	37.2 ± 11.8	38.6 ± 12.7	40.0 ± 14.5
BMI	27.5 ± 3.6	28.2 ± 10.8	33.9 ± 10.5
Sex	13/4	7/3	4/1

Age (yrs) and BMI (kg/m^2^) presented as Mean ± Standard error of the Mean (SEM) and sex as male/female

The study was approved by Norwegian Regional Committee for Medical and Healthcare Research Ethics (REK#384,542).

### Subjective and objective temperature exposure

In a cross-sectional fashion, we asked all the seafood handlers about cold exposure-related symptoms they may have experienced in connection with their work (Fig. 1B). A multi-item questionnaire assessing thermal working conditions, based on standard subjective judgement scales (ISO 10551), was used to collect data on thermal perception and self-reported cold exposure-related symptoms and background information. The questionnaire was presented to all participants, self-administered under supervision during scheduled breaks, and collected at the end of their shift (Supplement Methods). The separate group of non-shift day-working personnel employed in the administration of the same two plants also answered the questionnaires: 8 male, 4 female; age (mean ± SEM) 46.4 ± 12.9.

A subsample of participating Thawers, Packers, Operators, and Controllers agreed to carry a backpack equipped with thermal loggers (HiTemp 140, SN05142, MadgeTech), which recorded at 2-min intervals, to monitor the ambient temperature of their working area. Furthermore, each participant was equipped with an iButton (8K, DS1922L-F5#, Thermochron) device affixed to the skin of their upper arm, and such that brachial skin temperature data was recorded every 2 min.

### Plasma biomarkers analysis

Blood samples were collected from participating seafood handlers immediately before and after their work shifts, and plasma was subsequently isolated for analysis as described earlier [43]. Due to individual differences in shift start and end times, blood sampling, carried out sequentially by a single examiner, varied in time of day by up to 4.5 h for pre-shift samples and up to 6.5 h for post-shift samples. Multiplex Luminex kits were used to measure IL1α, IL6, IL10, TNF and IL12p70 (discovery assay, BioTechne); FGF21 and GDF15 (Merck). Samples were diluted 1:2 as recommended, and the procedure followed the manufacturers’ protocols. Analysis was performed on an xMAP Intelliflex instrument or MAGPIX multiplex reader (Luminex Corp.), and quantified with Quantist software (version 1.0, BioTechne) and Bio-Plex Data Pro software (Bio-rad Laboratories), respectively. Blood samples were also collected from the administration workers, however, only at the beginning of the day.

### Thermography

Infrared imaging was used to measure hand surface temperature of the seafood handlers [44]. Thermographic images were acquired with a Thermal Imaging Camera (T1020, FLIR Systems), with an emissivity coefficient of 0.95. For imaging, both hands were placed palms down on a plate of Styrofoam fixed to a scaffold at waist height. Images were taken immediately as the participants passed in and out through the doorway between the cold production area and the changing/resting area. Timestamps on the image files were used to determine the timing and length of the breaks; breaks lasting for more than 50 min were excluded. Images were quantified with ResearchIR MAX 4 software (Version 4.40.9.30, FLIR Systems). Region of interest (ROI) was defined as the entire surface of both hands (Figure S1) drawn manually on a tablet (Microsoft Surface Pen and Pro 10 tablet). Histograms with the temperature distribution of the thermal pixels in each ROI were extracted and exported to Excel file and rounded to the nearest whole degrees Celsius. Delta temperature after work bouts (before breaks) and after breaks were calculated, and the recovery rate during breaks (℃/min) was calculated (Figure S2-S3).

### Statistics

Questionnaire data were analysed by the Chi-square test. Ambient and skin temperature of participants in morning, evening, and night shifts were analysed by one-way ANOVA with Tukey correction. Two-way ANOVA statistical test with Tukey correction was used to study effects on FGF21, GDF15, cytokines and hand thermography. The association between age and plasma FGF21 and GDF15 was tested by linear regression analyses. Data is presented as Mean ± standard error of the Mean (SEM), if not otherwise stated. *P* < 0.05 is considered as significant.

## Results

Among the shift-working seafood handlers, 13% reported experiencing "White Fingers", while "Eczema/dry red skin in the face" was reported by 10%. None in the control group, the non-shift Administration workers, reported such symptoms. Both seafood handlers and administration workers reported "Pain or numbness in hands/fingers", 23% and 18%, respectively, and differences were not statistically significant (*P* = 0.76).

When asked about their working environment, 60% of the seafood handlers reported feeling cold at work (sometimes or often), whereas 33% of administration workers provided the same response (Fig. 1C). Interestingly, 25% of the administration workers reported discomfort due to cold environment while working, whereas this was reported by 12% of seafood handlers. Specifically, the attribution of cold discomfort to hands, feet and face among seafood handlers was 44%, 21%, and 17%, respectively, whereas among administration workers, it was 25%, 38%, and 13% (Fig. 1C). In addition, the workers were asked whether they felt warm during their working hours, and there was a statistically significant difference in the response between the seafood handlers and administration workers (*P* = 0.017). Specifically, 63% of the seafood handlers reported feeling warm at work, whereas 25% of individuals in the administration group reported the same (Fig. 1C). Of the seafood handlers 19% reported discomfort due to the warm work environment (Fig. 1C) and follow up on specific responses (Table S1).

**Fig. 2:**
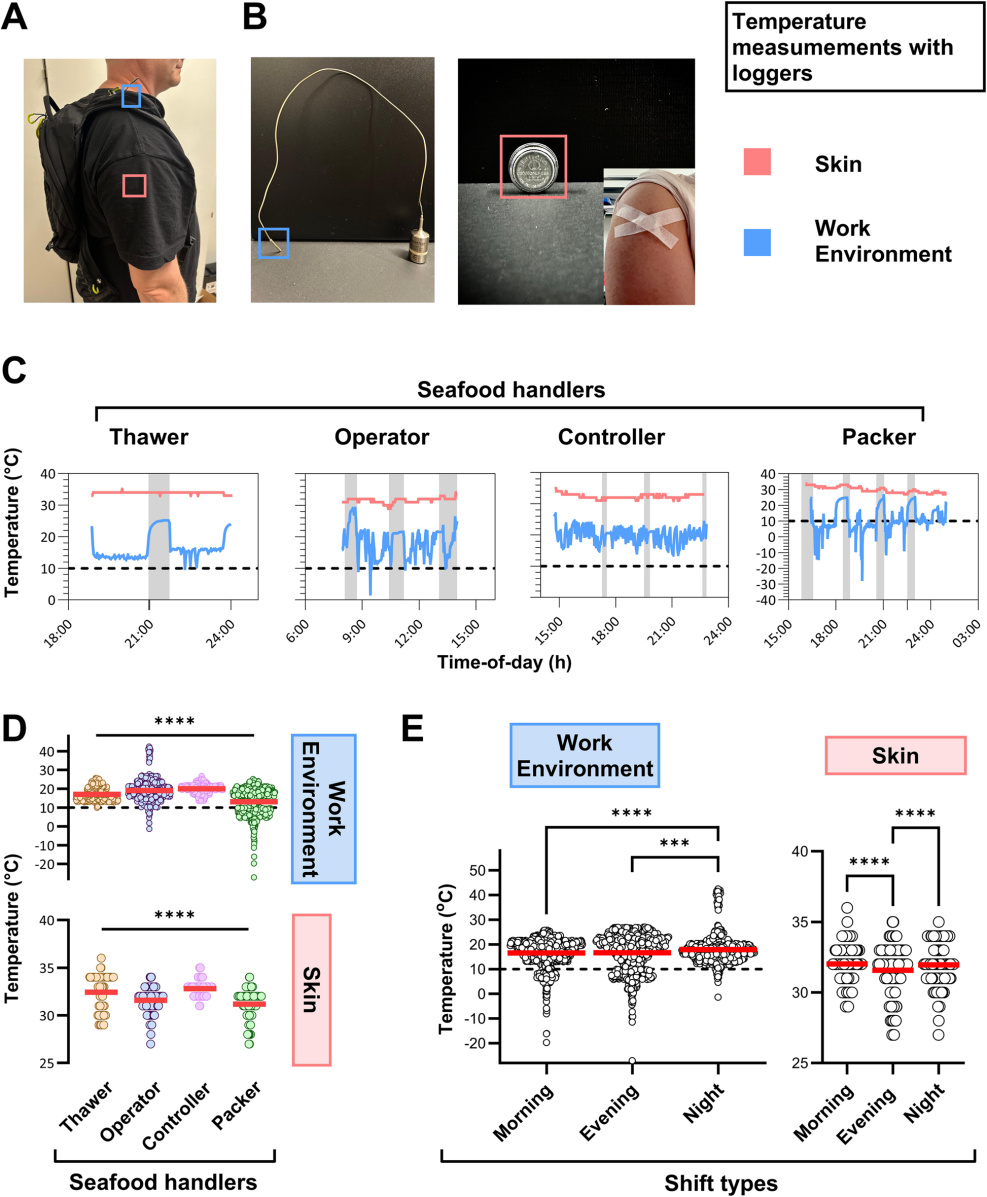
Thermal exposure associated with specific work tasks and shifts among seafood handlers. **A** A personal logger, carried in a backpack, recorded work environment temperature at a supraclavicular position, while an iButton, taped to the skin, measured brachial skin temperature. **B** Image shows personal thermal loggers that were used. **C** Examples of ambient temperature of the working environment (blue line) and brachial skin temperatures (red line) recorded using loggers carried by individual seafood handlers working as thawer, operator, controller, and packer. Shaded time intervals indicate breaks outside the production area. The dotted line indicates the limit of cold working conditions defined as ≤ 10 ℃. **D** Pooled ambient and skin temperature data of all seafood handlers. **E** Work environment temperature recorded during morning, evening, and night shifts, skin temperatures of the corresponding shift workers. Mean values are indicated. Statistical significance was tested by one-way ANOVA; ***P < 0.001, ****P < 0.0001

Objective measurements of cold exposure were conducted for various seafood handler tasks, including Thawers, Operators, Controllers, and Packers, using personal thermal data loggers over the course of a single work shift (Fig. 2A and B), examples are shown (Fig. 2C). Cold working conditions ≤ 10 ℃ were observed for the work tasks performed by Packers, Thawers and Operators, whereas the work environment temperature for Controllers stayed above this threshold (Fig. 2D; Figure S4). The ambient working temperature for Packers was recorded as the coldest (Fig. 2D). Furthermore, compared to other work tasks, Packers experienced the widest span in ambient working temperatures during their shifts, ranging from 25 ℃ to −27 ℃ (Fig. 2D). Brachial skin temperature monitored underneath the clothes ranged between 27 ℃ to 36 ℃ for all participants and somewhat followed the environmental temperature (Fig. 2D). Pooled ambient temperature data stratified according to task showed that mean thermal exposures were significantly different at 17.0 ℃, 19.1 ℃, 20.0 ℃, and 13.1 ℃ for Thawers, Operators, Controllers, and Packers, respectively. Stratification of ambient temperature according to shift types revealed that the participants in morning and evening shifts were exposed to significantly lower mean ambient temperature compared to night shift (Fig. 2E). Skin temperature, stratified by task, showed significantly different mean temperatures: 32.4 ℃, 31.6 ℃, 32.8 ℃, and 31.2 ℃ for Thawers, Operators, Controllers, and Packers, respectively (Fig. 2D). The lowest mean skin temperature was measured during the evening shift (Fig. 2E).

**Fig. 3:**
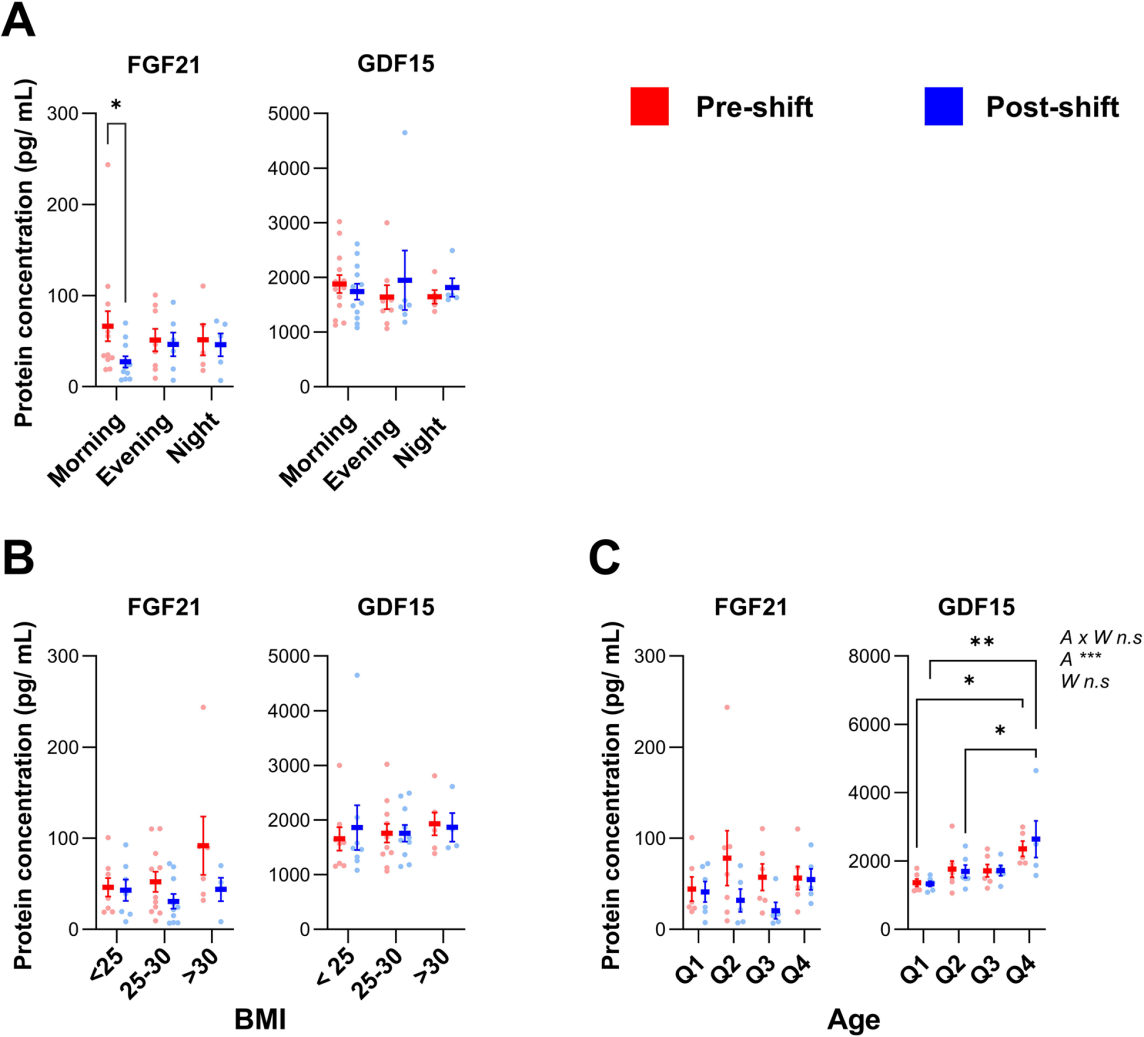
The effects of shift types on the plasma levels of putative cold exposure biomarkers. **A** FGF21 and GDF15 plasma levels in seafood handlers were analysed pre-shift (left) and post-shift (right) for different shift types (morning, evening, night). **B** FGF21 and GDF15 data stratified based on Body Mass Index (BMI) categories: normal weight (< 25 kg/m^2^), overweight (≥ 25 kg/m^2^ and < 30 kg/m^2^) and obesity (≥ 30 kg/m^2^); and **C**) Age quartiles: Q1 (21–27 yrs), Q2 (29–36 yrs), Q3 (39–52 yrs), Q4 (53–64 yrs). Statistical tests by two-way ANOVA; pre/post sampling (W), age (**A**) as independent factors; statistical significance is indicated as *P < 0.05; **P < 0.01.; ***P < 0.001. Plots show individual values with Mean ± SEM

**Fig. 4:**
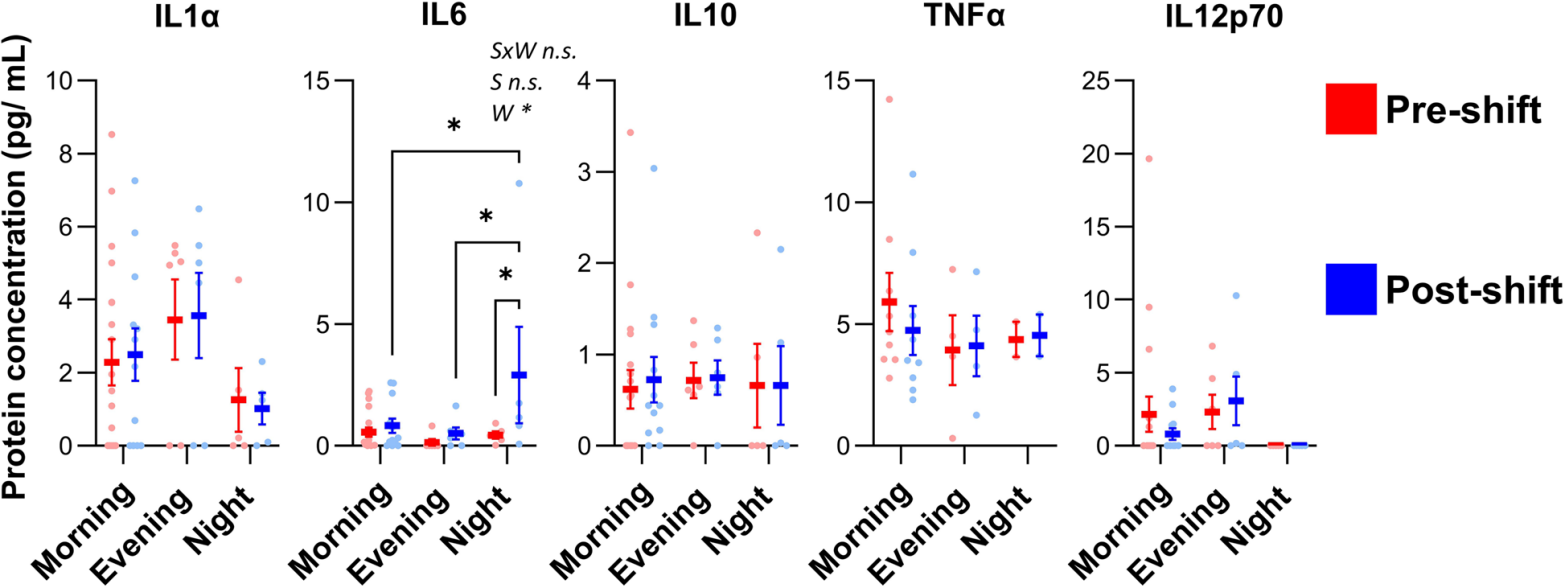
The effects of shift types on the plasma levels of inflammatory biomarkers. Panels show the effects of shift types (morning, evening, and night) on plasma levels pre- and post-shift of the cytokines IL1α, IL6, IL10, TNF, IL12p70. Two-way ANOVA was used with shift types (S) and pre/post sampling (W) as independent factors. Statistical significance is indicated as *P < 0.05. Data is presented as Mean ± SEM

Potential biomarkers of cold exposure were analysed: FGF21 and GDF15. The hypothesis was that the different shift types would impact the plasma concentrations of these hormones. We detected a statistically significant reduction in plasma levels of FGF21 among the seafood handlers during morning shifts (*P*-value < 0.05) but no changes during evening or night shifts (Fig. 3A). GDF15 plasma concentrations did not change during any of the shift types (Fig. 3A). BMI did not impact pre- or post-work levels of FGF21 or GDF15 (Fig. 3B), nor did age (Fig. 3C). However, age had a pronounced impact on basal plasma levels of GDF15 (*P*-value < 0.0001) but not FGF21 (Fig. 3C). There was a statistically significant interquartile difference in the plasma GDF15 levels in pre-shift conditions among participants in Q1 and Q4 (*P*-value = 0.0309, Fig. 3C), and linear relationship between age and GDF15, whereas there was no linear relationship between age and FGF21 (Figure S5) Inflammation markers in plasma were examined to investigate possible influence of shift types. Among seafood handlers, plasma IL6 levels increased during night shifts (*P*-value = 0.0054; Fig. 4B), whereas levels of IL1α, IL10, TNF and IL12p70 remained unchanged for all shift types (Fig. 4). Plasma measurements were also conducted on morning samples collected from the group of administration workers; FGF21 and GDF15 levels were not significant different in the administration group as compared to the seafood handlers (Figure S6). As quantifiable cytokine data were limited in the administration samples, they are not shown.

**Fig. 5:**
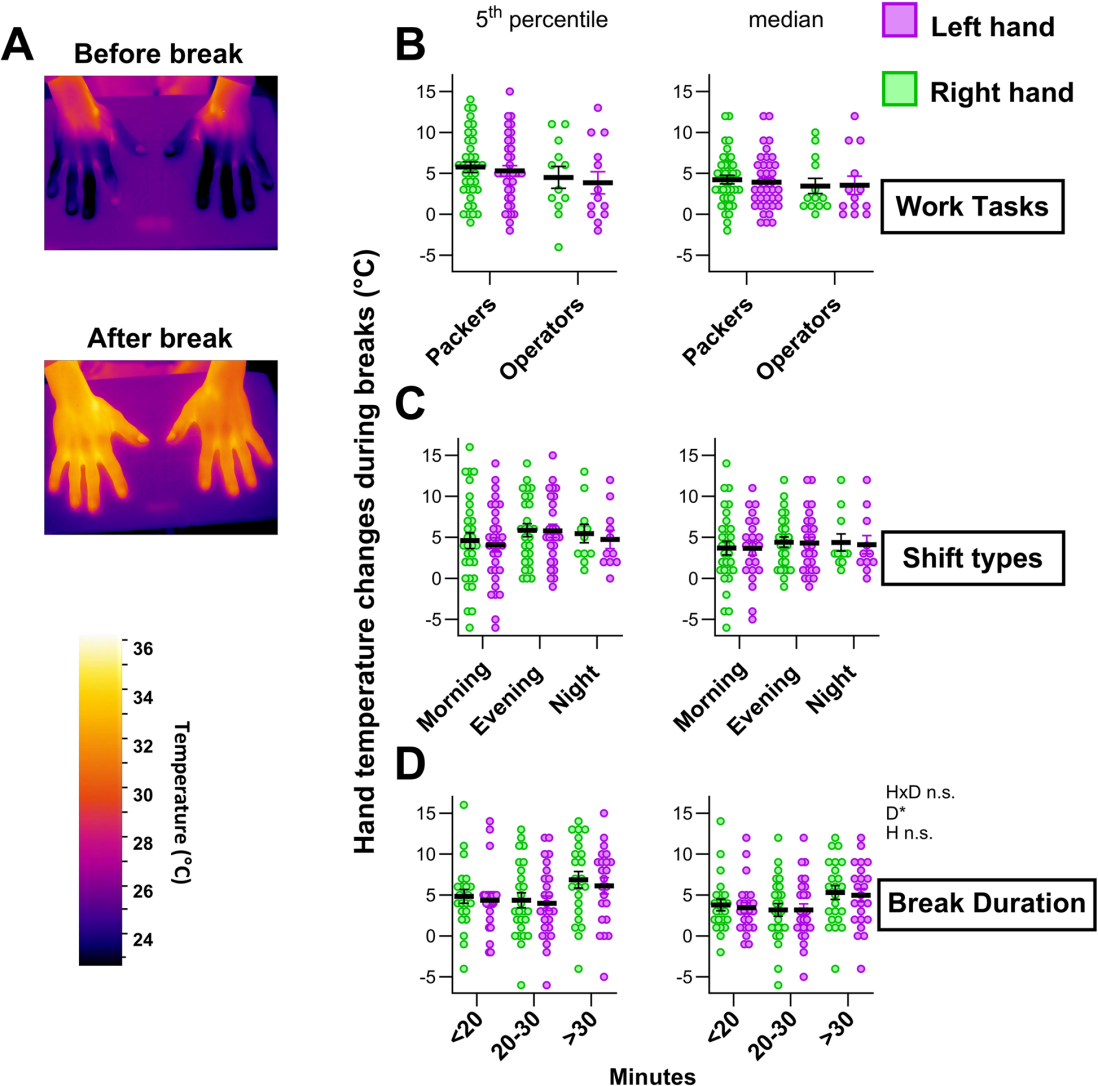
Change in hand temperature of participants between work bouts, during breaks. The hand temperature of participants was measured using infrared imaging before and after each break during one shift. **A** Representative images show the hand temperature of an individual after a work bout in the Packing area (upper image) and immediately after a 28-min break, going back to work (lower image). **B** Hand temperature changes associated with different work tasks; **C**) shift types; and **D**) break durations. The median and 5^th^ percentile temperature pixel values were identified before and after each break. Each dot represents one break-associated temperature change for participants’ left and right hands, as indicated. Data was collected for all breaks those participants had during one shift. Black bars show Mean ± SEM of overall break- related temperature change. Statistical significance was tested by two-way ANOVA with temperature change as the dependent factor; and left/right hand (H), break duration (**D**) as independent factors (HxD indicates the interaction). Statistical significance is indicated as *P < 0.05

**Fig. 6:**
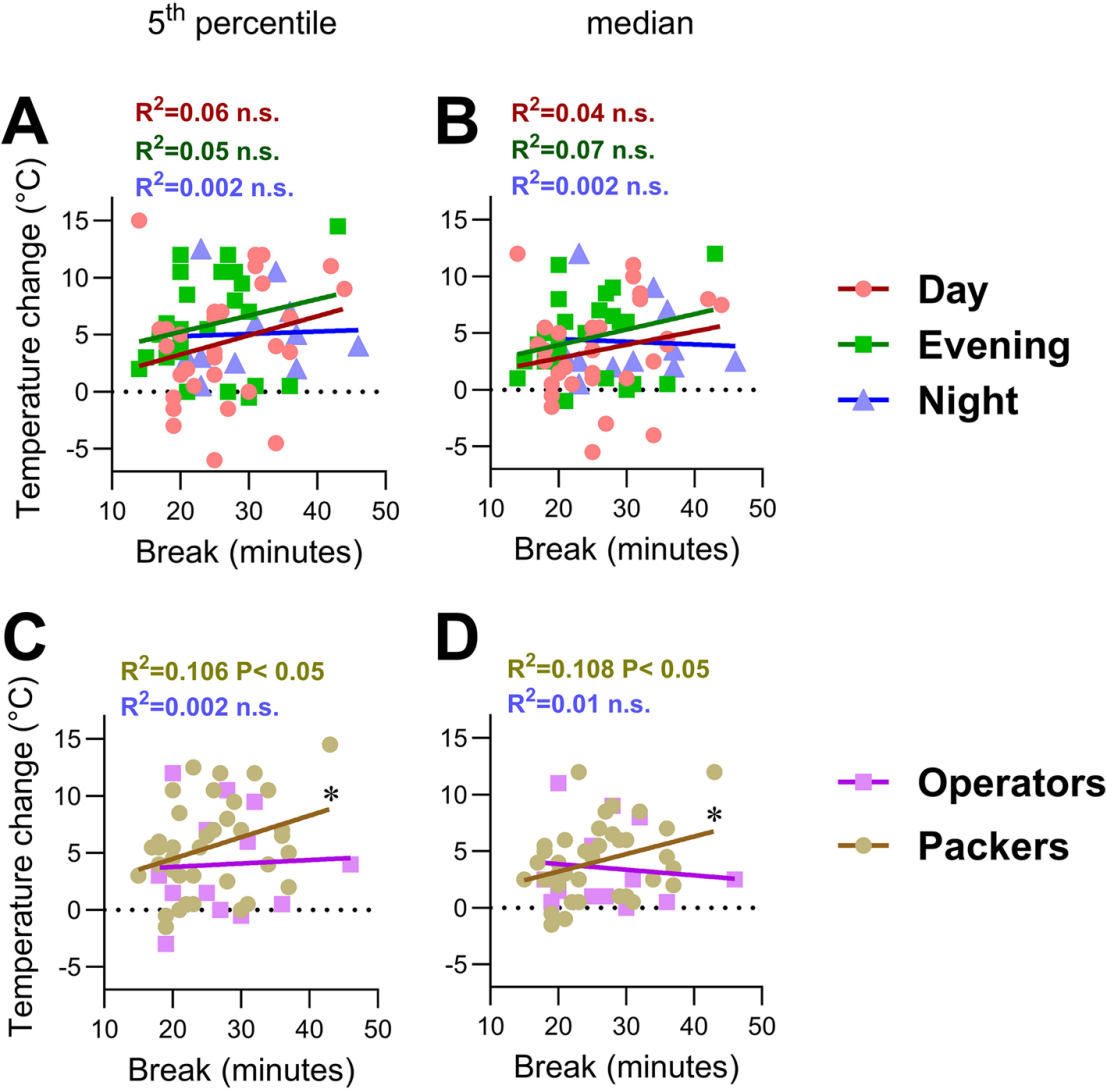
Hand temperature changes pre-post break in relation to break duration. Plots present regression lines to demonstrate the relationship between break duration and hand 5th percentile temperature change associated with **A**) different shifts and **C**) different work tasks. Corresponding **B**) shift data and **D**) task data for median temperature changes are shown. Each dot represents the mean temperature changes of both right and left hands for each individual break. R^2^ values indicate the variability of the data. Statistical significance is indicated as *P < 0.05

Hand temperature reflects thermoregulation in response to environmental cold exposure. We hypothesized that the hand temperature of the seafood handlers could be influenced by shift types, work tasks, and break durations. Hand thermography (Fig. 5A) showed no association in temperature changes of the hand related to work tasks (Fig. 5B) or shift types (Fig. 5C). Break duration had a significant impact on median (50th percentile) hand surface temperature changes (Fig. 5D). A similar and significant result was observed when considering only changes in the lower temperature extremes, the 5th percentile of pixels (Fig. 5D). The left and right hands did not respond differently, suggesting that the responses were non-lateralized. During night shifts, temperature recovery of the hands appeared less dependent on break duration, than during morning and evening shifts (Fig. 6A-B). For the Packers, but not Operators, the pre-post hand temperature significantly correlated with break duration, but not shift type (Fig. 6C-D). The thermography data also showed no significant correlation between hand recovery rates and shift types or work tasks (Figure S7).

## Discussion

This study examined cold exposure and thermoregulatory responses among shift-working seafood handlers, providing a comprehensive analysis of subjective reports and objective measurements. The results show significant variations in exposure levels and thermoregulatory responses based on specific tasks, and shifts, pointing out the complex interaction between work demands and organisational factors, and thermal comfort and recovery. The findings also shed light on how circadian rhythms and shift work influence inflammatory markers and metabolic markers, contributing to our understanding of how cold exposure may impact workers’ health.

Most seafood handlers reported experiencing both cold and warmth during work, confirming the presence of thermal strain in this occupation. These findings are consistent with previously documented symptoms associated with cold environments, such as white fingers and skin-related issues [26]. Subjective assessments further indicated that cold exposure particularly affected the hands, highlighting the extremities as a primary site of thermal discomfort in this occupational group. Seafood handlers tended to report experiencing both colder and warmer sensations at work compared to administrative personnel. This apparent paradox may be explained by the nature of their working conditions: while seafood handlers are exposed to a cold environment, they typically wear insulated clothing and engage in physically demanding tasks. The combination of external cold exposure and internal heat generation may contribute to subjective fluctuations between feeling excessively cold and overly warm throughout the workday.

Longitudinal temperature measurements of the work environment revealed an association between specific roles and tasks with varying levels of cold exposure, which was most severe during morning and evening shifts compared to night shifts. Our data show that the subdivisions Packers, Thawers, and Operators were exposed to ambient temperature below 10 ℃ during their work shift, whereas Controllers were not. Brachial skin temperature, measured under clothing, partially mirrored the ambient temperature of specific tasks, suggesting that the clothing did not fully counterbalance the low temperatures. Packers, that frequently move in and out of cold storage while handling the finished product, experienced the widest range of ambient temperatures and were more exposed to temperatures below 10 ℃ than any other work task group.

Our study also revealed that, in these factories, time of day influenced the extent of cold exposure, with ambient temperatures being lowest during the morning and evening shifts. Nevertheless, brachial skin temperature measurements were lowest during the evening shift alone. This disparity may indicate a difference in the physiological response to cold exposure depending on the time of day. Further investigation is warranted to clarify this relationship.

FGF21, a potential metabolic plasma marker for cold exposure, decreased from pre- to post-morning shift in seafood handlers, while remaining unchanged across other shifts. On the contrary, earlier studies have shown increased plasma FGF21 with cold exposure in both humans [40, 45] and rodents [34, 46, 47]. It is known that circulating FGF21 has been shown to peak in the morning, possibly due to increased levels of circulating fatty acids during nocturnal fasting, and then gradually decrease until noon [48], which is in line with our data. However, experimental studies have shown that cold exposure selectively increases FGF21 levels in the evening [40], a finding not supported by our study. This discrepancy may be attributed to differences in the population studied, as participants in the earlier studies were younger and leaner than those in our study [40, 45]. Given that FGF21 is recognised as a metabolic regulator, and elevated FGF21 levels are associated with obesity [49, 50], we stratified our FGF21 data by BMI and age. However, as our data suggest, no significant differences in FGF21 plasma levels were observed pre-and post-works across these groups.

Plasma GDF15 levels remained unchanged across all shift types in our study, a finding consistent with previous research in both mice [51] and in humans [40]. Campderros et al. demonstrated that the exposure of mice to 4 °C for 1 day dramatically increased GDF15 transcripts levels in BAT, reaching levels like those in the liver, but did not translate into altered plasma levels [51]. Furthermore, Hoekx et al. showed that neither cold exposure in the morning nor in the evening affects plasma GDF15 levels in humans [40]. It is worth noting that GDF15 has a short half-life of 3 h, which could make it difficult to detect any effects in our study population [52]. Although low temperature does not seem to impact plasma levels, we observed a potential age-related influence on circulating GDF15. Several earlier studies have presented evidence indicating that the expression of GDF15 is significantly lower in healthy and young individuals [53, 54]. The mechanisms responsible for the increase in circulating GDF15 levels with age are not fully understood. GDF15 has been shown to be increased in obese mice and humans [55, 56], but was not associated with BMI in our study.

Regarding inflammatory cytokines, IL6 increased significantly during the night shift but remained stable during morning and evening shifts. This is noteworthy because both circadian rhythm and environmental conditions influence IL6 production. A similar effect on IL6 has been reported in studies of mice [57] and humans [58]. Cold exposure of humans induced an increase in plasma IL6 after 1–2 h [58]. Hence, the increase in IL6 in night-shift workers could be potentially explained due to the cold exposure and circadian disruption. In contrast, the other cytokines measured did not change across the shifts. Processing of seafood can release bioaerosols into the air. Thus, bioaerosols and other airborne allergens may present a potential bias, as these can affect the levels of cytokines such as IL1β, IL6, IL10, and TNF [59].

In summary, our data provide preliminary evidence that certain biomarkers may reflect physiological responses to occupational cold exposure and shift work among seafood handlers. FGF21, a proposed marker of cold-induced metabolic regulation, decreased from pre- to post-morning shift, while remaining stable across other shifts. This may indicate a shift-specific thermoregulatory or metabolic adaptation, possibly related to the timing and intensity of cold exposure during early-day operations. In parallel, plasma IL-6 levels increased during night shifts Together, these findings suggest a selective inflammatory and metabolic response to night work and cold exposure, highlighting IL-6 and FGF21 as potential biomarkers of physiological strain in this occupational context. Further studies are needed to determine whether these responses reflect acute adaptation or contribute to longer-term health risks associated with shift work and cold environments.

Thermal imaging pre- and post-breaks provided valuable insight into peripheral thermoregulation at different shifts. We captured thermographic images of the seafood workers’ hands pre- and post- breaks to assess hand temperature and evaluate how breaks affect temperature recovery, serving as a proxy for thermoregulatory efficiency. The results showed that shift type—whether morning, evening, or night—does not seem to significantly affect hand temperature change during breaks. This aligns with the biomarker data, where only selective responses, such as decreased FGF21 during morning shifts and elevated IL-6 during night shifts were observed. Together, these findings indicate that while systemic markers may reflect shift- and context-specific physiological strain, peripheral thermoregulation remains largely robust across shifts in seafood handlers.

Break duration had a significant impact on hand temperature recovery; this suggests that implementing tailored break duration emerges as a critical factor for thermal recovery. Different countries have various regulations that typically entitle workers to 20–30 min breaks, but our results indicated that longer breaks may be more beneficial, particularly for tasks with higher cold exposure, such as Packers. Our data were limited, and further investigations are needed to reveal how work task may influence hand temperature recovery during breaks. As only smaller parts of the hands may become cold and stay cold during recovery, we compared developments of the lowest (5th percentile) and median temperature during breaks. The two methods of hand temperature analysis yielded similar results. Thermoregulatory responses, such as vasodilation and vasoconstriction, play crucial roles in maintaining skin temperature, and these responses are potentially influenced by circadian rhythm [60, 61] and anthropometric parameters such as age, BMI and sex [62, 63]. For example, it has been suggested that those who are obese or have a high BMI have higher hand skin temperature compared to those with normal weight or BMI [64].

This study has notable strengths and limitations. A key limitation involves the "healthy worker effect" (HWE), which may influence the findings [65]. Cold exposure can cause discomfort, prompting individuals to adjust their occupational or workplace settings to mitigate adverse effects. Consequently, our study population may exclude individuals particularly susceptible to shift work or cold exposure. Secondly, the relatively young age of participants may limit generalizability to older population, and it is plausible that workers struggling with either shift work or cold exposure may transition to alternative employment as they age. This focus on younger workers could influence health outcomes and affect the interpretation of results [65]. Over the past decade, considerable efforts have been made to understand sex-based differences in cold response. While variations in body shape and composition result in minimal disparities in thermoregulatory responses between men and women at the whole-body level [66–68], the limited number of women in our study precludes a comprehensive analysis of potential sex-based differences. This represents an important area for future research.

Despite being situated in the Arctic, the factories benefit from a rather stable climate due to the influence of the North Atlantic Drift. In this area, July is the warmest month, with an average temperature of 14.3 °C, while January experiences the coldest temperatures of the year, with an average minimum temperature of −4 °C [69]. Consequently, the workers in these factories encountered cold weather not just while working but also during their leisure activities. Research has shown that repeated cold exposure allows the body to acclimate or acclimatize in various ways depending on the duration and intensity of the cold exposure [70–73], which could reduce the effects of a cold working environment on the workers. In addition to ambient air temperature, several factors, such as the quantity and quality of protective clothing worn, can influence a worker's thermal balance, as hand temperature also would be influenced by the insulation properties of gloves used [74].

This study examines cold exposure among shift-working seafood handlers, assessed through both subjective reports and objective measurements. The findings indicate that exposure levels are strongly associated with specific tasks and shifts, with thermoregulatory responses differing based on time of day and with the type of task. This variation is evident in the thermal recovery of hands during breaks, highlighting the interplay between work demands and recovery dynamics. Although FGF21 and IL-6 exhibited shift-specific changes, these did not appear to reflect overlapping physiological responses. The findings of this study underscore the importance of tailoring workplace policies to address the specific needs of different worker groups. For seafood handlers, improving thermal conditions, providing appropriate clothing, optimizing break duration, and ensuring access to warm or cool areas, as needed, can help mitigate the negative impacts of temperature extremes [75]. Future research should explore the interplay between shift work, circadian biology, and thermal strain using longitudinal and mechanistic designs.

## Supplementary Information

Supplementary Material 1.

## Data Availability

No datasets were generated or analysed during the current study.
