# Supplementary material for: Impact of cold exposure on shift working seafood handlers in Northern Norway: a comparative analysis across work shifts
Source: J Occup Med Toxicol. 2025 Jun 23;20:22. doi: 10.1186/s12995-025-00469-2 (PMC12183870; doi:10.1186/s12995-025-00469-2)
Supplement: Supplementary file 1 — Supplementary Material 1. [file 12995_2025_469_MOESM1_ESM.docx]

**SUPPLEMENT MATERIAL**

**Supplement Methods**

***Questionnaire***

In this paper, we present responses to the following inquiries:

1. "Do you feel cold at work?". The possible answers were "Yes, sometimes/ Yes, often/ No". If the answer was "Yes, sometimes/ Yes, often", they were asked to answer either "Yes" or "No" to the extra set of statements about their perception of the ambient working temperature, such as "I often have cold hands", "I often have cold feet", "My face often feels cold", "I am so cold that I am shivering", "Cold work environment often causes discomfort".
2. "Do you feel warm at work?", The possible answers were "Yes, sometimes/ Yes, often/ No". In case the answer was "Yes, sometimes/ Yes, often", the follow-up statements were "I am often so warm that I am sweating" and "Warm work environment often causes discomfort".
3. Work-related symptoms experienced in the week before the survey, such as "Pain/ numbness in hands/fingers; White fingers; Eczema/dry red skin in the face ".


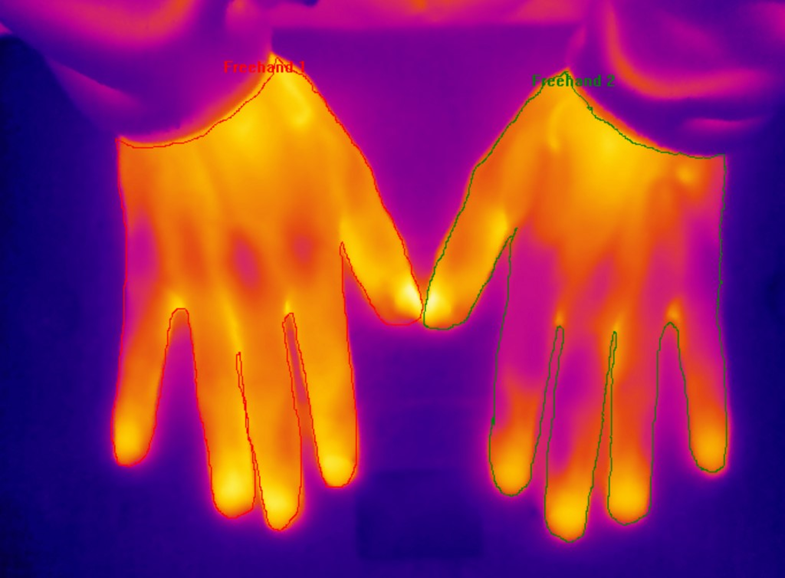


Figure S1. Demonstration of how Region of Interest (ROI) was marked on thermographic images.

***Calculations used on Thermography images***

5^th^ and 50^th^ percentile pixels calculation procedure

INDEX = (A§:A§, MATCH(SUM(D§:D§)*0.05, E§:E§, 1)) +1 (1)

A§:A§ - *temperature values*

D§:D§ - *pixel values*

E§:E§ - *the cumulative sum of pixel values*

To break it down, "(SUM(D§:D§)*0.05" calculates 5% of the sum of the pixel values in column D. "MATCH(SUM(D§:D§)*0.05" uses the 'MATCH' function to find the position of the value calculated in the first step (5% of the sum of the pixel values) with a range of cells in column E – the cumulative sum of pixels values. The "1" argument indicates that it will work and that it will look for the largest value that is less than or equal to the lookup value in ascending order. It is worth noting that the "MATCH" function has three types of match modes (-1, 0, 1). However, it is not possible to use the first two modes because the "-1" argument only works in the lookup array that has been sorted in descending order; however, the cumulative sum is calculated in the ascending one. The "0" argument finds the value that is exactly equal to the lookup value; however, the 5%- or 50%-pixel values are not presented in the cumulative sum array. The "INDEX" function returns the position value found by the "MATCH" function to a temperature value in column A. Unfortunately, the founded temperature by the "INDEX" function does not reflect the desired temperature because less than 5%- or 50% pixels represent the founded temperature. To fix this weakness, the authors used "+1" to ensure that 5% or 50% pixels reflected the desired temperature. The below figures visualized the calculations in 5% and 50% pixels.


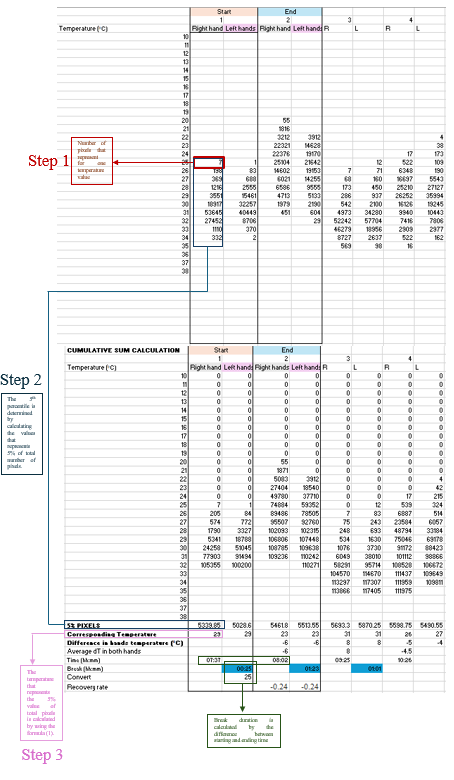


Figure S2. The orders of calculations the 5^th^ percentile pixels in the selected ROIs.


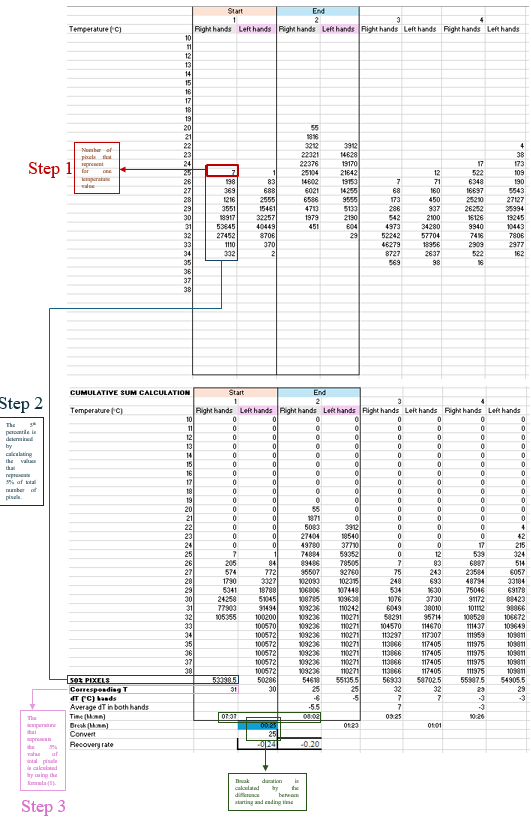


Figure S3. The orders of calculations the 50^th^ percentile pixels in the selected ROIs.

**Supplement Results**

**Table S1.** The difference in response among Administration and seafood handlers.

| Questions | Administration | Production | P-value |
| --- | --- | --- | --- |
| Do you feel cold at work? | 33% | 60% | 0.0999 |
| If yes, which of the following describes your work situation best? |  |  |  |
| I often have cold hands. | 25% | 43.75% |  |
| I often have cold feet. | 37.50% | 20.83% |  |
| My face often feels cold. | 12.50% | 16.67% |  |
| I am often so cold that I am shivering. | 0 | 6.25% |  |
| Cold work environment often causes discomfort. | 25% | 12.5% |  |
| Do you feel warm at work? | 25% | 63% | 0.0172 |
| If yes, which of the following describes your work situation best? |  |  |  |
| I am often so warm that I am sweating. | 0 | 81.25% |  |
| Warm work environment causes discomfort. | 25% | 19% |  |
| Work-related symptoms |  |  |  |
| Pain or numbness in hands/ fingers | 18.2% | 22.6% | 0.7600 |
| White fingers. | 0 | 13.3% | 0.2024 |
| Eczema/dry red skin in the face. | 0 | 9.6% | 0.2843 |
| Note. The percentages show the positive responses in each question | | | |

Figure S4. **The overview of work environment and skin temperature of the seafood handlers**. The backpack containing thermal loggers, used to measure the ambient working environment was carried by Thawers, Operators, Controllers, and Packers, as indicated by the colours. Additionally, an iButton on the arm was used to record brachial skin temperature. Red lines indicate mean temperature.

Figure S5. **The relationship between plasma levels of FGF21/ GDF15 and age in pre- and post-shift conditions.** *Refer to the legend of Figure 3. A-B) and C-D) show the plasma levels of FGF21 and GDF15 in the pre- (left, RED) and post- (right, BLUE) conditions, respectively.* Each plot presents the R^2^ values indicating the variability of the data, P-value demonstrates whether the association is statistically significant. Each dot represents the plasma levels of one individual. *Statistical significance is indicated as ^*^P ≤ 0.05.*

Figure S6**. Plasma levels of putative cold exposure biomarkers at baseline in administration workers and seafood handlers.** FGF21 and GDF15 plasma levels in seafood handlers and administration workers were analysed in the morning; pre-morning shift and pre-work, respectively. Groups were not significantly different.

Figure S7**. Average hand temperature recovery rate for different break durations.** (Hand temperature recovery versus break duration, categorized by shift types (A-B) and work task (C-D). Each plot presents the regression line equations to demonstrate the correlation between break duration and hand temperature recovery; R^2^ values indicate the variability of the data, P-value demonstrates whether the association is statistically significant. Each dot represents the average hand temperature recovery rates of both hands over time.
